# Fistula between the abdominal aorta and a retroaortic left renal vein: a rare complication of abdominal aortic aneurysm

**DOI:** 10.1590/0100-3984.2016.0070

**Published:** 2017

**Authors:** Leonor Garbin Savarese, Henrique Simão Trad, Edwaldo Edner Joviliano, Valdair Francisco Muglia, Jorge Elias Junior

**Affiliations:** 1 Faculdade de Medicina de Ribeirão Preto da Universidade de São Paulo (FMRP-USP), Ribeirão Preto, SP, Brazil; 2 CEDIRP - Central de Diagnóstico Ribeirão Preto, Ribeirão Preto, SP, Brazil

**Figure 1:**
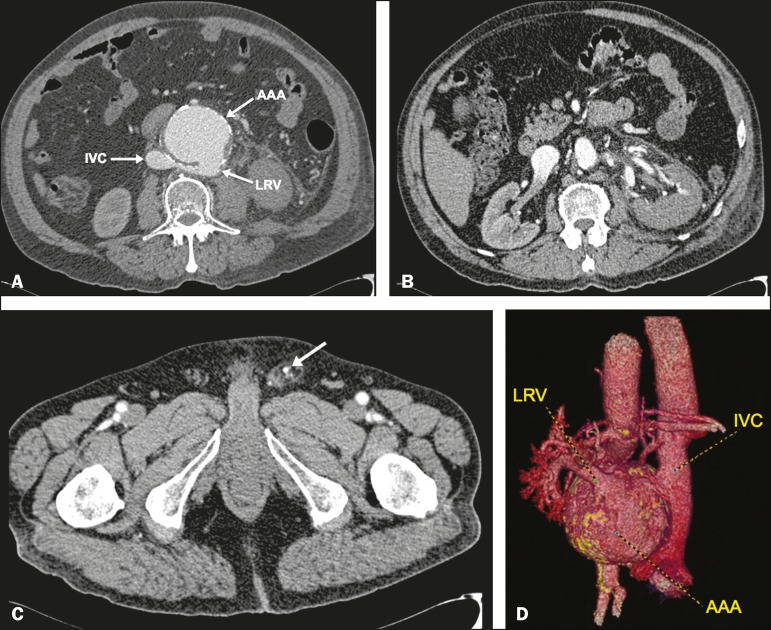
**A-C:** Contrast-enhanced axial CT slices showing equal opacification of the infrarenal aorta, a retroaortic left renal vein (LRV), and the inferior vena cava (IVC), confirming the fistula between an abdominal aortic aneurism (AAA) and the aberrant left renal vein (**A**). Note the reduced contrast enhancement of the left kidney (b) with dilatation and arterial enhancement of the left gonadic vein (arrow, **C**), accompanied by left varicocele. **D:** Contrast-enhanced CT, with three-dimensional reconstruction, in a posterior view, showing the retroaortic left renal vein in communication with the abdominal aortic aneurysm.

*Dear Editor*,

A 63-year-old man was referred to our hospital with abdominal pain, left varicocele, hematuria, and acute kidney injury. Multislice computed tomography (CT) revealed a 7.8 cm infrarenal abdominal aortic aneurysm and no contrast enhancement of the left kidney (Figure 1A), as well as a retroaortic left renal vein and dilatation of the left gonadic vein (Figure 1B), together with simultaneous contrast enhancement of the aneurysm, inferior vena cava, and left renal vein, suggesting the presence of a fistula between the abdominal aortic aneurysm and the aberrant left renal vein (Figures 1C and 1D). Given the suitability of the aneurysm, we decided to perform endovascular repair. Exclusion of the aneurysm and the aorto-left renal vein fistula was achieved after successful deployment of a 26-14 × 165 mm bifurcated endoprosthesis with a 16-16 × 95 contralateral limb (Gore Excluder; W.L. Gore and Associates, Flagstaff, AZ, USA). After endovascular management, renal function initially improved. The patient presented intraoperative hypotension, and the postoperative course was complicated by brain ischemia. Unfortunately, the patient died 65 days after surgery due to multiorgan failure.

Abdominal aortic aneurysm with spontaneous aorto-left renal vein fistula is a rare but well-described clinical entity, usually accompanied by abdominal pain, hematuria, and a nonfunctioning left kidney^(1)^. In male patients, left varicocele may result from venous overload in the pampiniform plexus via the left gonadal vein^(2)^. A review of the literature revealed only approximately 30 other reported cases^(3-6)^. Aorto-left renal vein fistula is often seen in patients with a retroaortic left renal vein, an anatomical variant present in 1.0% to 2.4% of the population^(7)^. It has been postulated that the vein is compressed between the pulsating aneurysm and the vertebral bodies, leading to erosion of the vessel wall and fistula formation. Open repair is the recognized method of treating rupture of an abdominal aortic aneurysm into a retroaortic left renal vein. Endovascular treatment is an attractive modality because it is minimally invasive, given its capacity for rapid percutaneous arterial access and graft deployment, as well as, if necessary, balloon occlusion for vascular control, thus minimizing blood loss in comparison with open surgery^(8)^. To our knowledge, this is the sixth reported case in which endovascular repair of this type of fistula has been attempted.
